# The Role of Mesenchymal Stromal Cells in the Treatment of Rheumatoid Arthritis

**DOI:** 10.3390/cells13110915

**Published:** 2024-05-25

**Authors:** Estera Bakinowska, Aleksandra Wiktoria Bratborska, Kajetan Kiełbowski, Maciej Ćmil, Wojciech Jerzy Biniek, Andrzej Pawlik

**Affiliations:** 1 Department of Physiology, Pomeranian Medical University, 70-111 Szczecin, Poland; esterabakinowska@gmail.com (E.B.); kajetan.kielbowski@onet.pl (K.K.); cmilmaciej@gmail.com (M.Ć.); wojciech.biniek6@gmail.com (W.J.B.); 2 Department of Internal Medicine, Poznań University of Medical Sciences, 60-780 Poznan, Poland; aleksandrabratborska@gmail.com

**Keywords:** rheumatoid arthritis, mesenchymal stromal cells, fibroblast-like synoviocytes, macrophages, inflammation

## Abstract

Rheumatoid arthritis (RA) is a chronic inflammatory joint disease characterised by the formation of a hyperplastic pannus, as well as cartilage and bone damage. The pathogenesis of RA is complex and involves broad interactions between various cells present in the inflamed synovium, including fibroblast-like synoviocytes (FLSs), macrophages, and T cells, among others. Under inflammatory conditions, these cells are activated, further enhancing inflammatory responses and angiogenesis and promoting bone and cartilage degradation. Novel treatment methods for RA are greatly needed, and mesenchymal stromal cells (MSCs) have been suggested as a promising new regenerative and immunomodulatory treatment. In this paper, we present the interactions between MSCs and RA-FLSs, and macrophages and T cells, and summarise studies examining the use of MSCs in preclinical and clinical RA studies.

## 1. Introduction

Rheumatoid arthritis (RA) is a chronic inflammatory joint disease associated with reduced quality of life, a decline in physical function, and an increased risk of developing comorbidities [1]. In recent decades, the burden of RA has increased, and this trend is expected to continue in the coming years [2]. The pathogenesis of RA is complex and involves interactions among numerous cells in inflamed joints, including fibroblast-like synoviocytes (FLSs), macrophages, and T cells, among others. These interactions induce invasive phenotype FLSs, which create a hyperplastic pannus. Furthermore, extensive inflammation in the synovial tissue eventually enhances cartilage and bone damage. Joint tissues are degraded by enzymes that damage the extracellular matrix (ECM), including matrix metalloproteinases (MMPs) and members of the disintegrin and metalloprotease with thrombospondin type 1 motif (ADAMTS) family. Multiple treatment agents are currently in use for RA patients. Disease-modifying antirheumatic drugs (DMARDs) are grouped into several classes, including conventional synthetic drugs (csDMARDs), such as methotrexate (MTX) and leflunomide; biologic agents (bDMARDs), such as tumour necrosis factor α (TNF-α) inhibitors; and JAK inhibitors [3]. Despite the efficacy of these agents and strategies, novel treatment methods are greatly needed. In recent years, multiple studies have investigated the potential use of mesenchymal stromal cells (MSCs) in in vitro and in vivo experiments in the context of RA. This review aims to discuss the current evidence on the use of MSCs as a novel treatment strategy for RA. We present immunomodulatory mechanisms induced by MSCs that could suppress the pro-inflammatory environment in the inflamed synovium and invasive features of RA-FLSs.

## 2. Mesenchymal Stromal Cells—Sources and Regenerative Properties

Mesenchymal stromal cells represent a population of multipotent progenitor stromal cells characterised by their capacity for self-renewal. Upon exposure to the appropriate stimuli, MSCs can differentiate into various specialised cell lineages derived from the mesenchyme, such as osteocytes, chondrocytes, myocytes, tenocytes, and ligamentocytes [4,5]. The human zygote-stage embryo displays totipotent characteristics, enabling the generation of both embryonic and extraembryonic tissues [6]. In adulthood, stem cells play a pivotal role in the mechanisms of renewal and regeneration. The activation and recruitment of stem cells represent a critical stage in the reparative processes that follow instances of trauma [7]. The investigation of MSCs has been a prominent area of scientific inquiry primarily because of their potential therapeutic efficacy in treating immune-related conditions and supporting tissue regeneration [8]. Various categories of stem cells exist, including pluripotent, multipotent, oligopotent, and unipotent stem cells. Pluripotent stem cells exhibit the potential to differentiate into cells of all three germ layers while avoiding the formation of extraembryonic structures. These cells are observed in different stages throughout human development [6]. Multipotent stem cells can undergo differentiation and give rise to tissues that originate from a specific germ layer. Oligopotent stem cells give rise to multiple cellular subtypes within a defined tissue. Unipotent stem cells exhibit the most restricted differentiation capacity, undergoing successive divisions to generate a singular cell lineage [9].

Bone marrow-derived mesenchymal stromal cells (BM-MSCs) exhibit significant regenerative and immunomodulatory capabilities and have undergone extensive evaluation in clinical trials, demonstrating both safety and effectiveness in various diseases [10]. Adipose tissue serves as an abundant reservoir of mesenchymal stromal cells (AT-MSCs), which can be obtained through subcutaneous lipoaspiration [11]. AT-MSCs and BM-MSCs exhibit comparable morphological characteristics and cell surface markers, yet their differentiation potential varies. Xu et al. provided evidence that BM-MSCs and AT-MSCs exhibited specific capacities for differentiating into three different cell lineages, notwithstanding similarities in cell surface markers and phenotypic properties. BM-MSCs exhibited an enhanced osteogenic differentiation capacity while displaying a reduced adipogenic differentiation potential compared to AT-MSCs. Nonetheless, there is no disparity in the potential for chondrogenic differentiation between BM-MSCs and AT-MSCs [12]. However, the employment of umbilical cord or umbilical cord-derived blood as a reservoir of MSCs (UC-MSC) also exhibits considerable potential [13]. UC-MSCs exhibited enhanced cell multiplication rates, clone-forming ability, and anti-inflammatory capacity [14]. The dental pulp serves as a reservoir of MSCs with neurotropic characteristics, derived from the neural crest [15]. Interestingly, MSCs could be extracted from the synovium. This tissue serves as a reservoir of stem cells that can be used for the regeneration of defects within the articular cartilage [16]. MSCs derived from the synovium showcase a greater percentage and an enhanced capacity to differentiate into adipocytes and chondrocytes compared to MSCs derived from the bone marrow [17].

The immunomodulatory properties of MSCs are crucial in understanding the underlying mechanisms responsible for disrupting immune homeostasis. The immunomodulatory mechanisms of MSCs have been investigated by using both in vitro and in vivo experimental models of autoimmune conditions. MSCs can recruit and regulate T cells through both direct cell-to-cell contact and paracrine signalling pathways. The paracrine effects of MSCs are attributed to the release of various molecules, such as growth factors, cytokines with anti-inflammatory properties, chemokines, and extracellular vesicles containing bioactive components, such as non-coding RNA (ncRNA) [4,18,19,20]. Additional research has also demonstrated that extracellular vesicles released by stem cells (MSC-EVs) exhibit immunosuppressive and immunomodulatory characteristics [21,22,23,24]. They can enhance cell growth, prevent cell death, and decrease oxidative damage, thereby fostering cellular renewal and suppressing inflammation [25]. MSC-EVs contain functional proteins that contribute to their ability to modulate diseases. Qiu et al. examined the immunomodulatory characteristics of MSC-EVs and proposed that their protein composition is crucial in modulating the immune response. MSC-EVs can also enhance the secretion of immunoregulatory cytokines [22]. Consequently, the regenerative and immunomodulatory properties of MSCs and MSC-derived exosomes have been investigated in the context of numerous inflammatory conditions, including RA.

## 3. Mesenchymal Stromal Cells and Rheumatoid Arthritis Fibroblast-like Synoviocytes

**Figure 1:**
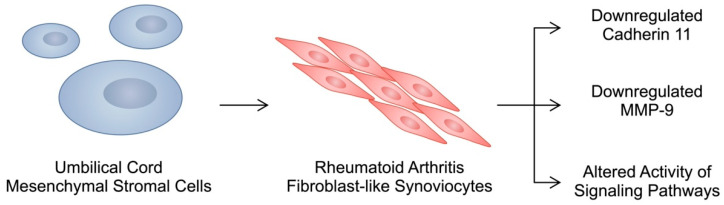
UC-MSCs have been found to induce various beneficial effects on FLSs that could be beneficial in RA, including a downregulated expression of cadherin 11 and MMP-9, as well as an altered activity of signalling pathways in FLSs.

RA-FLSs are one of the most significant cells in the pathogenesis of RA. They actively contribute to bone and cartilage degradation and the formation of a hyperplastic pannus, a key feature of RA. Furthermore, RA-FLSs interact with immune cells and stimulate pro-inflammatory responses [26]. In recent years, numerous studies have examined potential pathways and molecules associated with the invasiveness of RA-FLSs, which could be targeted to reduce their proliferation or migration [27,28,29]. Importantly, studies have found that stem cells also interact with RA-FLSs, which can induce various effects. They secrete immunomodulatory factors and extracellular vesicles containing bioactive cargo that alter the behaviour of RA-FLSs. To begin with, stem cells have been found to both contribute to and suppress RA progression. Both mechanisms involve cadherin 11 (CDH11), a protein that regulates the adhesion of surrounding cells. The co-culture of RA-FLSs with BM-MSCs was associated with the enhanced production of placental-induced growth factor, a pro-angiogenic molecule associated with increased FLS invasiveness and an enhanced secretion of IL-17 from T cells. This cell–cell interaction was found to occur through CDH11 [30,31,32]. By contrast, Zhao et al. reported that the co-culture of RA-FLSs with UC-MSCs could reduce the expression of cadherin-11 (CDH11) through the secretion of IL-10 [33]. CDH11 has been suggested to be involved in the pathogenesis of RA, as CDH-11-null mice show reduced arthritis activity [34]. IL-17, one of the pro-inflammatory cytokines involved in the progression of RA, increases the expression of CDH11 in FLSs [35]. Moreover, the engagement of CDH11 in RA synovial fibroblasts enhanced the expression of MMPs, thus contributing to cartilage damage [36]. The expression of CDH11 in peripheral blood was significantly greater in RA patients with active disease [37]. Therefore, the stem cell-mediated suppression of CDH11 could be associated with various beneficial mechanisms for alleviating the disease. Different results obtained by the above-mentioned studies implementing stem cells might result from different types of examined MSCs, as cells obtained from the umbilical cord are more similar to embryonic stem cells compared to BM-MSCs. Therefore, the regenerative and immunomodulatory potential of stem cells from various sources may differ [38]. The cell-to-cell contact of UC-MSCs with FLSs could reduce the invasive features of the latter cells. Moreover, it downregulates the expression of MMP-9, thus reducing the risk of tissue destruction [39]. The co-culture of UC-MSCs with FLSs has also been associated with an altered activity of signalling pathways. Specifically, UC-MSCs enhanced the expression of p53 and suppressed that of pAkt. Moreover, UC-MSCs improved the chondrogenic potential of FLSs [40] (Figure 1). In addition, studies demonstrated other beneficial mechanisms induced by UC-MSCs. As previously mentioned, MSCs can secrete exosomes with bioactive cargo, thus communicating with other cells. These extracellular vesicles may contain non-coding RNA, molecules that regulate gene expression. This RNA molecule family is broad and includes microRNA (miRNA), long non-coding RNA (lncRNA), and circular RNA (circRNA), among others. MiRNAs can bind to mRNA and suppress its translation, while lncRNA and circRNA can inhibit the activity of miRNAs and are known as miRNA “sponges”. Mi et al. found that stimulating RA-FLSs with exosomes obtained from UC-MSCs significantly alters the expression of 13 miRNAs. Importantly, these structures could suppress the proliferation of RA-FLSs [41]. Huang and collaborators demonstrated that the co-culture of RA-FLSs with exosomes obtained from UC-MSCs could increase the expression of miR-140-3p in the former cells. The authors demonstrated that miR-140-3p could inhibit the expression of glucocorticoid-inducible kinase 1 (GSK1). Importantly, these exosomes inhibited pro-inflammatory responses in RA-FLSs by decreasing the secretion of interleukin-1β (IL-1β) and TNF-α [42]. MiR-451a is another RNA molecule secreted by UC-MSCs that has been suggested to decrease the invasiveness of RA-FLSs [41]. Importantly, the treatment of MSCs with various factors can modulate their activity or immunomodulatory properties. Chiu and colleagues showed that tumour necrosis factor-related apoptosis-inducing ligand (TRAIL) is upregulated in UC-MSCs after stimulation with IL-1β. TRAIL stimulates apoptotic pathways, and culturing stimulated MSCs with IL-1β-stimulated RA-FLSs induced apoptosis in the latter cells [43].

Besides UC-MSCs, the influence of other types of stem cells on RA-FLSs has also been examined. The stimulation of RA-FLSs with BM-MSC-derived exosomes significantly alters the expression of 16 miRNA molecules, including 7 down- and 9 upregulated molecules [44]. Therefore, the bioactive cargo present in the vesicles can alter the expression of miRNAs in RA-FLSs to affect their behaviour. For instance, BM-MSC-derived exosomes were found to contain miR-320a, a molecule that targeted CXC chemokine ligand 9 (CXCL9). As a result, the activation, invasion, and migration of RA-FLSs were suppressed [45]. CXCL9 is a chemokine upregulated in the synovium of RA compared with osteoarthritis (OA); furthermore, its expression is positively correlated with the activity of RA [46,47]. Interestingly, plasma levels of CXCL9 were also positively correlated with the concentrations of N-terminal pro-B-type natriuretic peptide (NT-ProBNP), a biomarker of cardiac diseases [48]. MiR-34a is another RNA molecule present in extracellular vesicles obtained from BM-MSCs, which suppresses the proliferation of RA-FLSs. Mechanistically, Wu and collaborators determined that miR-34a could target and suppress the expression of cyclin 1, thus enhancing the p53 pathway [44].

The immunomodulatory and regenerative properties of MSCs can be improved through pretreatment or gene transfection. As these cells can secrete exosomes as carriers of bioactive cargo, transfection with an appropriate miRNA can generate exosomes overexpressing the required molecules. Several researchers have examined the impact of modified MSCs on RA-FLSs. Firstly, Chen et al. transfected BM-MSCs with miR-150-5p to obtain exosomes containing this miRNA molecule. These structures could suppress the migration and invasion of RA-FLSs. Furthermore, they downregulated the expression of MMP-14, as well as vascular endothelial growth factor (VEGF). Thus, these exosomes could suppress cartilage degeneration and angiogenesis, important processes in RA progression [49]. Similarly, exosomes containing miR-124a and miR-451a also reduced the invasiveness of RA-FLSs [41,50]. Another molecule that suppresses the invasive features of RA-FLSs is lncRNA heart and neural crest derivatives expressed 2-antisense RNA 1 (HAND2-AS1). The molecule was found to decrease the production of IL-6 and TNF-α by suppressing nuclear factor-κB (NF-κB) signalling, which is a major pro-inflammatory pathway. As previously mentioned, lncRNA can sponge miRNA, which alters gene expression. Mechanistically, HAND2-AS1 targets miR-143-3p, a miRNA that suppresses the expression of tumour necrosis factor alpha-inducible protein 3 (TNFAIP3). Therefore, HAND2-AS1 positively regulates the expression of TNFAIP3. The use of MSC exosomes overexpressing HAND2-AS1 decreased the invasiveness of RA-FLSs through the HAND2-AS1/miR-143-3p/TNFAIP3 pathway, which ultimately suppressed NF-κB signalling [51].

Importantly, apart from ncRNA, extracellular vesicles can be used as nanocarriers of other substances. Studies have demonstrated the beneficial role of curcumin in patients with RA. Specifically, a meta-analysis demonstrated that the use of curcumin was associated with improved laboratory parameters, such as C-reactive protein, as well as clinical parameters, including DAS28 [52]. He and colleagues loaded curcumin into exosomes obtained from MSCs and showed that these structures could reduce the viability of synovial cells stimulated with lipopolysaccharide (LPS). Moreover, the modified exosomes induced apoptosis and decreased the expression of pro-inflammatory mediators, as well as MMP1. Importantly, MSC-derived structures induced more significant effects than curcumin alone [53]. Taken together, multiple studies in the literature suggest that MSC or MSC-derived vesicles can suppress the invasive features of RA-FLSs. Table 1 summarises the interactions between MSCs and MSC-derived exosomes with RA-FLSs.

## 4. Mesenchymal Stromal Cells and Macrophages

Macrophages are found in large numbers in the inflamed synovial membrane; as a major source of TNF-α, they play a crucial role in RA progression [54]. Macrophages, along with activated fibroblasts, stimulate osteoclasts by contacting the ligand activator of the nuclear factor kB receptor (RANKL) with its receptor RANK, present on macrophages, dendritic cells, and preosteoclasts [55,56]. Classically, two subpopulations of macrophages are distinguished: the pro-inflammatory M1 and the anti-inflammatory M2 phenotypes. However, macrophages exhibit phenotypic and functional plasticity, which indicates that other phenotypes of these cells are present. Under the influence of microenvironmental factors, they can differentiate into forms similar to the M1 or M2 types [57,58,59,60,61]. M1 macrophages, by secreting cytokines, chemokines, and enzymes, exhibit extensive pro-inflammatory, destructive, and remodelling capabilities, contributing significantly to inflammation and joint deterioration during both the acute and chronic stages of RA [62]. Among the cytokines involved in lesion progression, TNF is most closely associated with RA pathogenesis, and its antagonists have been successfully used to treat the disease [63]. The most important cytokines secreted by macrophages include TNF-α, IL-1α, IL-1β, IL-6, granulocyte-macrophage colony-stimulating factor (GM-CSF), CXC chemokines, macrophage inflammatory protein (MIP), monocyte chemoattractant protein (MCP), MMPs, and neopterin [64,65,66].

These molecules have been found to contribute to the pathogenesis of RA. For instance, TNF-α is a pleiotropic cytokine that stimulates the synthesis of various molecules, such as other cytokines, adhesion molecules, prostaglandin E2, collagenase, and collagen in synovial cells. In RA, TNF-α is predominantly produced by macrophages residing in the synovial membrane and at the junction between cartilage and the pannus, playing a central role in the cascade of inflammation [63,64]. This cytokine promotes vasodilation and increases vascular permeability, facilitating the infiltration of lymphocytes, neutrophils, and monocytes into the affected area. Together with IL-17, TNF-α initiates the production of chemokines, including CXCL1, CXCL2, and CXCL5, which attract neutrophils. Moreover, it enhances the expression of cell adhesion molecules [67,68,69]. TNF-α and IL-1β trigger the activation of synovial fibroblasts, which then release RANKL and macrophage colony-stimulating factor 1 (M-CSF), crucial factors in osteoclast formation. This process upregulates osteoclast activity, exacerbating bone destruction [65,70]. Various experimental and clinical findings demonstrate the significance of TNF-α. The concentration of TNF-α in synovial fluid correlates with the presence of lining macrophages and the extent of radiographically assessed bone erosion [64]. G-CSF serves as the primary regulator of granulocyte production. It is synthesised by bone marrow stromal cells, endothelial cells, macrophages, and fibroblasts, and its production is induced by inflammatory stimuli. G-CSF functions through the G-CSF receptor (G-CSFR), which is expressed on early myeloid progenitors, mature neutrophils, macrophages, and endothelial cells. G-CSF enhances neutrophil production and release, mobilises hematopoietic stem and progenitor cells, and regulates the differentiation, lifespan, and functions of mature neutrophils. Additionally, G-CSF may impact macrophages by increasing their population, enhancing phagocytic activity, and modulating the production of inflammatory cytokines and chemokines [62,67,71,72,73].

MSCs influence macrophage polarisation, maintaining a balance between the pro- and anti-inflammatory states. Due to cell–cell interactions, MSCs reduced the production of TNF-α, IL-1β, and IL-6 in mouse macrophages that were stimulated with LPS [74]. MSCs consistently generate IL-6, which, in conjunction with LPS and pro-inflammatory cytokines, shifts pro-inflammatory M1 macrophages towards the anti-inflammatory M2 phenotype that produces IL-10. Additionally, anti-inflammatory macrophages release high levels of TGF-β1, which helps quell inflammation and facilitate tissue regeneration [75]. Macrophage polarisation is crucial for maintaining tissue regeneration and homeostasis. Multiple studies have shown that exosomes derived from MSCs encourage the transition from M1 to M2 polarisation, elevating levels of anti-inflammatory cytokines and chemokines. This mechanism effectively reduces inflammation [59,60,76,77,78,79]. As previously mentioned, macrophages can contribute to the process of osteoclastogenesis. MSCs were found to inhibit the RANKL-induced formation of osteoclasts, thereby preventing systemic bone loss [80,81].

## 5. Mesenchymal Stromal Cells and T Cells

Several subtypes of T cells and the interactions between them, other immune cells, and RA-FLSs have been widely studied in the context of RA. They are the main initiators of immune responses that contribute to cartilage and bone destruction. Dendritic cells (DCs) and other antigen-presenting cells stimulate CD4+ T cells to differentiate into various helper T cells (Th), such as Th1, Th22, and Th17, with the latter playing a crucial role in joint inflammation by secreting interleukin (IL)-17 and IL-22 [82,83,84]. Both cytokines contribute to FLS proliferation and stimulate the secretion of TNF-α, IL-1β, IL-6, monocyte chemoattractant protein 1 (MCP1), and macrophage inflammatory protein-3 (MIP3A), among others [84,85]. Pro-inflammatory cytokines stimulate FLSs to damage the surrounding tissues in joints, as well as promote the release of RANKL, a major stimulator of osteoclast proliferation [86]. Furthermore, T follicular helper (Tfh) cells and T peripheral helper (Tph) cells stimulate B cells to produce autoantibodies, including rheumatoid factor (RF) and anti-citrullinated peptide antibodies (ACPAs) [85]. In addition, T cells are another major source of RANKL [87]. Importantly, the population of regulatory T cells (Tregs) is depleted in RA patients, which leads to a lower secretion of anti-inflammatory cytokines and an impaired immune tolerance against autoantigens [88]. Tregs produce IL-10, IL-35, and transforming growth factor (TGF)-β and therefore inhibit the proliferation of effector T cells [89]. In RA patients with a lower percentage of Tregs, the inhibitory effect of these cytokines is diminished. The imbalance between Th17 and Tregs is one of the core factors that contribute to the development and progression of RA [90].

Inflammation intensity is a direct modulator of the healing efficacy of MSCs [91]. Pro-inflammatory cytokines, such as IL-1, IL-17, TNF-α, and IFN-γ, lead to the activation of immunomodulatory mechanisms of MSCs, stimulating them to secrete extracellular vesicles (EVs). Furthermore, MSCs regulate the immune response via cell–cell interactions with macrophages, B cells, T cells, and NK cells [92].

Allogenic BM-MSCs, as well as MSC-differentiated chondrocytes, suppress T-cell activation and proliferation induced by type II collagen (CII). Furthermore, they inhibit the secretion of IFN-γ and TNF-α by Th cells [93]. Another study showed that mouse BM-MSCs augment Treg differentiation, inhibit the secretion of TNF-α and IFN-γ, and increase the production of anti-inflammatory IL-10. Additionally, they suppress the excessive differentiation of Th1 and Th17 cells [94]. Tatara et al. demonstrated that BM-MSCs can inhibit the proliferation of Th17, partially acting through prostaglandin E2 (PGE2) and indoleamine 2,3-dioxygenase (IDO); however, they did not influence Tregs [95]. As IDO is an important enzyme responsible for tryptophan metabolism, its increased concentration leads to impaired function and promotes apoptosis in T cells [96]. The crucial role of IDO and IL-10 in the immunosuppressive activity of MSCs has been confirmed in a study in which BM-MSCs from healthy donors were epigenetically modified to have upregulated IDO and IL-10 expressions. The modified cells more effectively inhibited the proliferation of CD 4+ T cells and their differentiation towards Th17 than MSCs without modifications [97]. Mouse BM-MSCs with overexpressed programmed cell death 1 ligand (PD-L1) reduced the percentage of Th1 and Th17 but enhanced that of Tregs. PD-L1-transfected mouse BM-MSCs had lower concentrations of pro-inflammatory cytokines, including IL-1β, IL-6, IL-17, IFN-γ, and TNF-α, while levels of anti-inflammatory IL-10 were increased [98]. BM-MSCs reduce the number of CD4+ and CD8+ T cells, thereby reducing the levels of IL-2, IL-6, IL-9, IL-17, IFN-γ, and TNF-α. Moreover, BM-MSCs can regulate T cell functions by upregulating the mRNA expression of IL-4, IL-10, and TGF-β [99].

BM-MSCs and BM-MSC-derived EVs increase the population of Tregs and decrease the production of numerous pro-inflammatory cytokines [100]. Similarly, EVs released by UC-MSCs inhibit T cell proliferation and their differentiation into Th17, while increasing the number of Tregs. Consequently, they contribute to higher levels of TGF-β and IL-10, as well as lower IL-17 [101]. Exosomes released by UC-MSCs restored the balance between Tregs and Th17 in collagen-induced arthritis (CIA) mouse models, simultaneously decreasing the number of Th1 and the concentration of IL-6, IL-17, and TNF-α. Moreover, levels of TGF-β and IL-10 were significantly increased [102]. Moreover, UC-MSCs inhibit the proliferation and differentiation of Tfh, partially through the secretion of IDO in the presence of IFN-γ at the site of inflammation. This inhibition was observed both in vitro and in vivo in CIA mouse models [103]. Apart from suppressing T cell differentiation, UC-MSCs decrease the concentrations of TNF-α, IFN-γ, IL-2, IL-4, and IL-17, as well as enhance the population of Tregs, which in turn contribute to higher levels of TGF-β. Additionally, granzyme B and perforin, lytic enzymes produced by CD8+ T cells that contribute to the development of RA, are significantly lower after incubation with UC-MSCs [19]. Intriguingly, UC-MSC therapy was suggested to be as successful as MTX in hindering the proliferation of T cells and increasing their apoptosis in CIA rats. Additionally, it restores the balance between Th17 and Treg, and their related cytokines [104]. Moreover, UC-MSCs allow us to achieve similar therapeutic outcomes in CIA mice as the injection of anti-TNF or anti-CD20 antibodies in reducing the number of Th1 and Th17 cells, as well as in increasing the number of Tregs. Interestingly, among the three types of therapies, only UC-MSCs cause a decrease in Tfh percentages, as well as a downregulation in IL-6 secretion. Additionally, they decrease levels of IFN-γ, IL-1β, and IL-17 [105]. Importantly, endoplasmic reticulum (ER)-stressed UC-MSCs secrete more prostaglandin E2 (PGE2) and consequently inhibit Tfh more effectively [106]. In active RA patients, the combination of DMARDs and UC-MSCs is safe and effective in lowering TNF-α and IL-6, as well as increasing the number of Tregs [107].

AD-MSCs can elevate the Treg-to-Th17 ratio up to twofold and therefore alter the cytokine profile by increasing levels of TGF-β and decreasing the secretion of TNF-α, IL-17, and IL-21 [108]. Another study showed that AD-MSCs are able to upregulate GATA-binding protein-3 (GATA3) and forkhead box P3 (FOXP3), transcription factors of Th2 and Treg, respectively [109]. Th2 secretes anti-inflammatory IL-4 and IL-13, which suppress the progression of arthritis [110]. Contrastingly, they downregulate T-box transcription factor TBX21 (T-bet) and retinoid-related orphan receptor γt (RORγt), transcription factors of Th1 and Th17, respectively [109]. AD-MSCs delay the onset of CIA in rats, as well as augment Treg proliferation and their IL-10 production [111].

Dental follicle stem cells (DFSCs) increase the number of Tregs and suppress their apoptosis in vitro. Moreover, stem cells decrease TNF-α secretion and stimulate IL-10 production [112]. Interestingly, human dental pulp stem cells (DPSCs) overexpressing hepatocyte growth factor (HGF) increase the number of Tregs more effectively than normal DPSCs [113]. HGF is increased in the synovium of RA patients and promotes inflammation and bone destruction in joints [114]. Dong et al. showed its multifunctional effects in RA, since in the early phases of the disease, it inhibited its progression by increasing the number of immunomodulatory Tregs. In the late phase, however, HGF activated FLSs and contributed to the elevation of proinflammatory IL-6 [113].

Another two trials on refractory RA patients explored the effects of autologous BM-MSCs and showed a decrease in the Th17 percentage and IL-17 levels, as well as a significant increase in Tregs and their cytokines, specifically IL-10 and TGF-β. Moreover, patients had a higher expression of FOXP3 [115,116]. However, allogeneic expanded AD-MSCs did not significantly influence the T-cell profile in patients suffering from refractory RA [117] (Table 2).

## 6. Preclinical and Clinical Studies

As demonstrated in the previous paragraphs, MSCs induce several beneficial immunomodulatory mechanisms that could be associated with suppressed RA. Therefore, studies have also examined the efficacy of MSCs in preclinical in vivo and clinical studies. To begin with, the previously discussed cells were also investigated in preclinical settings and showed important beneficial effects in RA animal models [39,42,43,44]. Moreover, the previously mentioned MSC-derived exosomes containing ncRNA molecules could also suppress the progression of arthritis in animal models [45,49]. Nevertheless, more studies investigated the use of MSC/MSC-derived exosomes in RA animal models. Significant efforts have been put into the examination of UC-MSCs. Liu et al. studied the efficacy of BX-U001, a product from UC-MSCs in CIA mouse models. The authors administered the MSCs through tail vein injection. The cellular treatment significantly improved clinical scores compared with those of a cell-free control group. Importantly, at the end of this study, the histological scores of animals treated with UC-MSCs were not significantly different from those of the healthy group, highlighting the promising efficacy of BX-U001. Furthermore, cellular therapy resulted in a significant decrease in IL-6 concentrations [118]. Additionally, Vohra and colleagues demonstrated that UC-MSCs significantly suppressed paw swelling, and the beneficial effects were observed even 6 weeks after infusion [19]. Recently, beneficial effects were observed when animal models were treated with gingival-derived mesenchymal stromal cells and exosomes derived from these cells [119,120].

Studies also investigated different strategies for UC-MSC administration. For instance, three cycles of intravenous administrations of UC-MSCs every two weeks were also associated with improved clinical arthritis scores, reduced invasion of the pannus, decreased gene and protein expressions of IL-1β and IL-6, and an upregulation of anti-inflammatory IL-10 [121]. By contrast, repeated high-dose administrations of UC-MSCs were not associated with improved arthritis scores or ankle thickness in a CIA mouse model. Moreover, cellular treatment did not improve tissue degradation [122]. Thus, the beneficial effects of UC-MSCs might depend on the amount of stem cells introduced or the period of cellular treatment. Table 3 summarises the studies investigating the use of MSCs and MSC-derived exosomes in RA animal models. Interestingly, UC-MSCs were suggested to induce greater reductions in the levels of pro-inflammatory cytokines than MTX [123]. Moreover, the effects of UC-MSC treatment in CIA mice were similar to those of biological agents targeting TNF and CD20 [105].

The promising results of preclinical studies led to investigations of the efficacy and safety of therapies implementing MSCs in RA patients. Wang et al. examined the use of UC-MSCs in RA patients. Specifically, participants received MTX/leflunomide/hydroxychloroquine, together with a solvent with UC-MSCs. Importantly, after 3 months of treatment, the addition of stem cells significantly decreased DAS28 scores and reduced the concentrations of RF and CRP, as compared with patients who received DMARDs without UC-MSCs. Moreover, treatment with MSCs increased the population of Tregs in peripheral blood. Regarding the safety analysis, the cellular treatment did not induce major toxicities [107]. Recently, several clinical trials also examined the potential of UC-MSCs in RA patients. In a phase Ia clinical trial, a single intravenous injection of UC-MSCs significantly decreased the DAS28-ESR score after 4 weeks. However, there were no differences in swollen and tender joint counts nor pain VAS scores [124]. In a trial by Wang and colleagues, the authors analysed the long-term safety and efficacy of patients treated intravenously with UC-MSCs. The authors observed that the DAS28 and HAQ scores were significantly reduced 1 and 3 years after treatment compared to the pretreatment period. In addition, reduced levels of CRP and RF at these time points were found [125]. According to the clinicaltrials.gov website, two trials are registered with the status “not yet recruiting” and one with the status “recruiting”. These studies will examine the safety and efficacy of autologous AD-MSCs (intravenous infusion, 54 estimated participants) [126], BX-U001 (intravenous infusion, 16 estimated participants) [127], and allogeneic UC-MSCs (intravenous infusion, 20 estimated participants) [128], which indicates that future trials will increase the evidence on the potential use of MSCs in patients with RA in the future.

## 7. Conclusions

To conclude, the pathogenesis of RA strongly involves inflammatory responses. Numerous studies demonstrated that MSCs can interact with RA-FLSs and immune cells to suppress their invasive and pro-inflammatory features. MSCs can secrete immunosuppressive agents and exosomes containing bioactive cargo that suppress the expression of pro-inflammatory mediators. Furthermore, MSC-derived exosomes can be used as nanocarriers for the targeted delivery of required molecules, such as miRNA. Preclinical animal studies demonstrated that MSCs could significantly reduce clinical arthritis and histological scores, as well as reduce serum levels of pro-inflammatory cytokines. In addition, clinical trials demonstrated promising benefits of using MSCs in RA patients. Future studies should examine various strategies of MSC administration, together with the proper dose and combination with DMARDs.

## Figures and Tables

**Table 1:** Summary of the impact of MSCs or MSC-derived exosomes on the behaviour of RA-FLSs.

Stem Cells	Mechanism	References
UC-MSCs	UC-MSCs secrete IL-10 which reduces the expression of cadherin-11, which is implemented in the pathogenesis of RA.	[33]
	Co-culture of UC-MSCs with FLSs suppresses their invasive features and downregulates MMP-9 expression.	[39]
	UC-MSCs secrete exosomes that suppress pro-inflammatory responses and enhance apoptosis of RA-FLSs.	[42]
	IL-1β-stimulated UC-MSCs upregulated the expression of TRAIL, which then enhanced the apoptosis of RA-FLSs.	[43]
BM-MSCs	BM-MSC-derived exosomes contain miR-320a which targeted CXCL9, thus suppressing the migration and invasive properties of RA-FLSs.	[45]
	BM-MSC-derived exosomes contain miR-34a that targets and suppresses the expression of cyclin 1.	[44]
MSC-derived exosomes containing miR-150-5p	Exosomes overexpressing miR-150-5p suppressed RA-FLS migration and invasion, as well as downregulated the expression of MMMP-14 and VEGF.	[49]
MSC-derived exosomes containing miR-124a	miR-124a present in exosomes suppressed proliferation and enhanced the apoptosis of RA-FLSs.	[50]
MSC-derived exosomes containing miR-451a	Exosomes overexpressing miR-451a suppressed RA-FLS migration, proliferation, and invasion.	[41]
MSC-derived exosomes containing HAND2-AS1	Exosomes containing HAND2-AS1 could suppress the invasive features of RA-FLSs through the HAND2-AS1/miR-143-3p/TNFAIP3 pathway that ultimately inhibited NF-κB signalling.	[51]
MSC-derived exosomes containing curcumin	Exosomes loaded with curcumin could reduce the viability and downregulate pro-inflammatory cytokines in LPS-stimulated synovial cells.	[53]

MSCs—mesenchymal stromal cells; UC-MSCs—umbilical cord mesenchymal stromal cells; BM-MSCs—bone marrow mesenchymal stromal cells; RA-FLSs—rheumatoid arthritis fibroblast-like synoviocytes; NF-κB—nuclear factor-κB; miR—microRNA.

**Table 2:** Summary of studies investigating the impact of MSCs and MSC-derived extracellular vesicles on T cells.

Source of Stem Cells	Model	Results	Reference
BM-MSCs and MSC-differentiated chondrocytes	In vitro	Inhibited T cell activation, ↓ IFN-γ, ↓ TNF-α	[93]
Mouse BM-MSCs	In vitro and CIA mice	↑ Treg, ↓ Th1, ↓ Th17, ↓ IFN-γ, ↓ TNF-α, ↑ IL-10	[94]
Mouse BM-MSCs	In vitro	↓ Th17 partially through IDO and PGE2, no influence on Treg	[95]
Epigenetically modified MSCs	In vitro	↓ T cells, ↓ Th17, ↓ IL-17, ↓ IFN-γ	[97]
PD-L1-MBMMSCs	CIA mice	↑ Treg, ↓ Th1, ↓ Th17, ↓ TNF-α, ↓ IFN-γ, ↓ IL-1β, ↓ IL-6, ↓ IL-17, ↑ IL-10	[98]
BM-MSCs	In vitro	↓ T cells, ↓ IL-2, ↓ IL-6, ↓ IL-9, ↓ IL-17, ↓ IFN-γ, ↓ TNF-α, ↑ TGF-β, ↑ IL-4, ↑ IL-10	[99]
BM-MSCs and their EVs	In vitro	↑ Treg, ↓ IFN-γ, ↓ TNF-α, ↓ IL-4, ↓ IL-22	[100]
EVs from UC-MSCs	CIA mice	↓ T cells, ↓ Th17, ↑ Treg, ↓ IL-17, ↑ TGF-β, ↑ IL-10	[101]
UC-MSCs’ Exos	CIA mice	↓ Th1, ↓ Th17, ↑ Treg, ↓ TNF-α, ↓ IL-6, ↓ IL-17, ↑ TGF-β, ↑ IL-10	[102]
UC-MSCs	In vitro and CIA mice	↓ Tfh partially through IDO production	[103]
UC-MSCs	In vitro and CIA rats	↓ T cells, ↑ Treg, ↓ TNF-α, ↓ IFN-γ, ↓ IL-2, ↓ IL-4, ↓ IL-17, ↑ TGF-β, ↓ granzyme B, ↓ perforin	[19]
UC-MSCs	CIA rats	↓ T cells, ↓ Th17, ↑ Treg, ↓ IL-17, ↑ TGF-β	[104]
UC-MSCs	CIA mice	↓ Tfh, ↓ Th1, ↓ Th17, ↑ Treg, ↓ IL-1β, ↓ IL-6, ↓ IL-17, ↓ IFN-γ	[105]
ER-stressed UC-MSCs	In vitro	↑ PGE2, ↓ Tfh	[106]
UC-MSCs	Patients with active RA	↑ Treg, ↓ TNF-α, ↓ IL-6	[107]
AD-MSCs	In vitro	↑ TGF-β, ↑ Treg, ↓ Th17, ↓ TNF-α, ↓ IL-17, ↓ IL-21	[108]
AD-MSCs	In vitro	↑ FOXP3, ↑ GATA3, ↓ T-bet, ↓ RORγt, ↑ Treg, ↑ Th2, ↓ Th17, ↓ Th1	[109]
AD-MSCs	CIA rats	↑ Treg, ↑ IL-10	[111]
DFSCs	In vitro	↑ Treg, ↓ TNF-α, ↑ IL-10	[112]
DPSCs overexpressing HGF	CIA mice	↑ Treg, ↓ TNF-α, ↓ IL-6	[113]
autologous BM-MSCs	Patients with refractory RA	↑ TGF-β, ↑ IL-10, ↑ Treg, ↑ FOXP3, ↓ Th17, ↓ IL-17	[115,116]
AD-MSCs	Patients with refractory RA	No significant difference in T cells	[117]

MSCs—mesenchymal stromal cells; BM-MSCs—bone marrow mesenchymal stromal cells; UC-MSCs—umbilical cord mesenchymal stromal cells; AD-MSCs—adipose tissue-derived mesenchymal stromal cells; CIA—collagen-induced arthritis; Treg—regulatory T cells; TNF-α—tumour necrosis factor α; IL—interleukin; ↑—increased; ↓—decreased.

**Table 3:** Summary of the efficacy of MSCs or MSC-derived exosomes in RA animal models.

Cells/Structures	Animal Models	Effects	References
UC-MSCs/IL-1β-stimulated UC-MSCs	CIA mouse	Reduced arthritis score and paw thickness.	[43]
UC-MSC exosomes	CIA rats	Suppressed pathological feet, reduced serum levels of inflammatory mediators.	[42]
BM-MSC exosomes	Rats injected with Freund’s complete adjuvant F5881	Reduced feet swelling, inflammatory infiltration, and synovial hyperplasia.	[44]
UC-MSCs	CIA mouse	Cellular treatment eliminated immune infiltration and pannus formation.	[39]
Exosomes containing miR-150-5p	CIA mouse	Reduced arthritis score, paw thickness, and angiogenesis.	[49]
Exosomes containing miR-320a	CIA mouse	Reduced serum levels of pro-inflammatory mediators	[45]
BX-U001 (UC-MSCs)	CIA mouse	Cellular treatment decreased clinical and histological scores, as well as decreased the levels of IL-6.	[118]
UC-MSCs	CIA mouse	UC-MSCs significantly reduced paw swelling and the beneficial effects of cellular treatment were observed even 6 weeks after introduction of MSCs.	[19]
GMSCs	STIA mouse	Administration of GMSCs reduced inflammatory infiltration, paw thickness, and clinical scores, among others.	[119]
GMSC exosomes	CIA mouse	Treatment with exosomes reduced arthritis scores, together with decreased osteoclast activity, cartilage damage, and synovial hyperplasia.	[120]
UC-MSCs	CIA mouse	Three cycles of stem cells significantly improved clinical arthritis and histological scores, as well as reduced the expression of inflammatory mediators.	[121]
UC-MSCs	CIA mouse	Repeated high-dose cellular treatment improved neither arthritis score nor tissue damage.	[122]
UC-MSCs	CIA mouse	Decreased histological score similar to the biological agents targeting CD20 and TNF.	[105]

MSCs—mesenchymal stromal cells; UC-MSCs—umbilical cord mesenchymal stromal cells; CIA—collagen-induced arthritis; GMSCs—gingival-derived mesenchymal stromal cells; IL—interleukin; STIA—serum transfer-induced arthritis model.

## Data Availability

Not applicable.
